# From Friend to Enemy: Dissecting the Functional Alteration of Immunoregulatory Components during Pancreatic Tumorigenesis

**DOI:** 10.3390/ijms19113584

**Published:** 2018-11-13

**Authors:** Hui-Ching Wang, Wen-Chun Hung, Li-Tzong Chen, Mei-Ren Pan

**Affiliations:** 1 Graduate Institute of Clinical Medicine, College of Medicine, Kaohsiung Medical University, Kaohsiung 807, Taiwan; u106801006@kmu.edu.tw; 2 Division of Hematology and Oncology, Department of Internal Medicine, Kaohsiung Medical University Hospital, Kaohsiung Medical University, Kaohsiung 807, Taiwan; 3 National Institute of Cancer Research, National Health Research Institutes, Tainan 704, Taiwan; hung1228@nhri.org.tw (W.-C.H.); leochen@nhri.org.tw (L.-T.C.); 4 Division of Hematology/Oncology, Department of Internal Medicine, National Cheng Kung University Hospital, Tainan 704, Taiwan

**Keywords:** pancreatic cancer, immunotherapy, CTLA-4, PD-1, PD-L1

## Abstract

Pancreatic ductal adenocarcinoma (PDAC) is a lethal disease with a 5-year survival rate of approximately 8%. More than 80% of patients are diagnosed at an unresectable stage due to metastases or local extension. Immune system reactivation in patients by immunotherapy may eliminate tumor cells and is a new strategy for cancer treatment. The anti-CTLA-4 antibody ipilimumab and anti-PD-1 antibodies pembrolizumab and nivolumab have been approved for cancer therapy in different countries. However, the results of immunotherapy on PDAC are unsatisfactory. The low response rate may be due to poor immunogenicity with low tumor mutational burden in pancreatic cancer cells and desmoplasia that prevents the accumulation of immune cells in tumors. The immunosuppressive tumor microenvironment in PDAC is important in tumor progression and treatment resistance. Switching from an immune tolerance to immune activation status is crucial to overcome the inability of self-defense in cancer. Therefore, thoroughly elucidation of the roles of various immune-related factors, tumor microenvironment, and tumor cells in the development of PDAC may provide appropriate direction to target inflammatory pathway activation as a new therapeutic strategy for preventing and treating this cancer.

## 1. Introduction

Pancreatic ductal adenocarcinoma (PDAC) has very high mortality rate among cancers with less than 8% survival beyond 5 years [1]. As for the obscure clinical course of the disease, most patients have advanced stage and unresectable status at diagnosis. Metastatic PDAC has a dismal prognosis due to resistance to current therapy. Standard chemotherapy with gemcitabine shows modest effects with median overall survival (OS) ranging from 5 to 8 months [2,3]. Adjuvant chemotherapy with gemcitabine- or fluorouracil-based regimens improved the overall survival in resectable disease [4,5]. Combinations of chemotherapy have been reported with some benefit to survival in metastatic PDAC. For locally advanced and metastatic disease, polychemotherapy using FOLFIRINOX (folinic acid, 5-fluorouracil, irinotecan, oxaliplatin) improved the median survival to 11.1 months [6]. The phase III MPACT study showed that the median OS was significantly longer for nab-paclitaxel and gemcitabine compared to that for gemcitabine alone (8.7 vs. 6.6 months, hazard ratio (HR) = 0.72) [7]. However, the results are not satisfactory for clinical practice and the patients finally experience disease progression and recurrence.

Cancers develop multiple ways to escape immunosurveillance and process immunoediting [8]. The antitumor efficacy of the immune checkpoint blockade was displayed by blocking the downstream regulators of immunity including cytotoxic T-lymphocyte antigen 4 (CTLA-4) and programmed cell death 1 (PD-1) or its ligand, programmed cell death ligand 1 (PD-L1). CTLA-4, also known as CD152, is a co-inhibitory molecule expressed on CD4+ CD25+ regulatory T cells, and binds with its ligands B7.1 and B7.2 expressed on antigen-presenting cells to halt immune response and reinforce signaling for cell cycle progression [9]. CTLA-4 blocking antibodies such as ipilimumab and tremelimumab arouse immune detection and stimulate T cell activation [10,11]. PD-L1, also known as CD274, expressed on antigen-presenting cells, binds to PD-1 (known as CD279) expressed on activated T cells, B cells, NK cells, and monocytes [12,13]. PD-L1 is generally considered to influence the immune response at later stages in peripheral tissues [14,15]. Targeting the PD-L1/PD-1 pathway enhances T cell proliferation and production of interferon-γ (IFN-γ), tumor necrosis factor-α, and IL-2, and is directly correlated with a regressive response in tumor size [16,17]. Anti-PD-1 antibodies such as pembrolizumab and nivolumab contribute remarkably to treating different cancers including metastatic melanoma, non-small cell lung carcinoma, renal cell carcinoma, urothelial carcinoma, Hodgkin’s lymphoma, microsatellite instability-high (MSI-H) or mismatch repair-deficient solid tumors, and head and neck carcinoma [18,19,20,21]. Therefore, immune checkpoint blockade has revealed a promising benefit in treating a range of cancer types.

PDAC comprises a massive stroma and few tumor cells, which build a natural barrier and defense against immune cells and conventional cytotoxic agents [22]. The stroma containing extracellular matrix (ECM), fibroblasts, pancreatic stellate cells, immunoregulatory cells, and endothelial cells makes up 90% of the PDAC components. The remaining 10% components are cancer cells. Thus, the microenvironment mainly obstructs the therapeutic effects of drugs, in contrast to most other solid tumors. However, the microenvironment is dynamic and change continuously during pancreatic tumorigenesis from pre-neoplastic pancreatic intraepithelial neoplasm (PanIN) to invasive PDAC. In the meta-analysis, positive PD-L1 expression was highly correlated with poorer overall survival and pathologic differentiation in PDAC patients [23]. However, the relationship between PD-L1 and PDAC patients remains inconclusive. In this review, we focus on the functional transitions of associated immunoregulatory cells and their interplay during cancer development and treatment. 

## 2. The Pancreatic Cancer Microenvironment

Abundant stroma forms a natural barrier leading to a hypoxic surrounding and accumulation of immunosuppressive cells and cytokines, which characterize pancreatic cancer with low immunogenicity and non-inflamed circumstances. Pancreatic cancer displays an immunosuppressive microenvironment that is immunologically ignorant and poorly infiltrated by T lymphocytes [24].

### 2.1. Pancreatic Stellate Cells-Stromal Fibroblast

During the development of chronic pancreatitis, activated cancer-associated fibroblasts (CAFs) derived from pancreatic stellate cells (PSCs) contribute to fibrosis and produce massive ECM during transformation to PDAC [25]. A prior study demonstrated that activated PSCs encompassed human PanIN, suggesting that PSCs participate in the development of early-stage PDAC [26].

Multiple cellular pathways have been associated with regulating CAFs function. For example, hypoxia-inducible factor-1 alpha and galectin-1 induce the expression of sonic hedgehog (SHH) and Gli 1 to activate fibroblasts leading to impedance of blood perfusion and a growth advantage to tumor cells [27,28,29,30,31,32]. Another key player between CAFs and cancer cells is the fibroblast activation protein (FAP), which mediates cancer cell invasion and cell cycle activation by Rb protein inhibition [33]. These evidences suggest that abundant stroma creates an additive factor for tumor advanced progression. However, a recent study indicated that myofibroblast depletion led to the remodeling of the tumor extracellular matrix without improving the efficacy of gemcitabine treatment. Notably, myofibroblast depletion resulted in tumors with a decreased effector and regulatory T cells (Teff/Treg) ratio and increased CTLA-4 expression. Myofibroblast depletion combined with anti-CTLA-4 antibodies significantly reduced the tumor burden in mice [34]. These evidences indicate that the cross-talk between cancer cells and stroma is sophisticated, but targeting the checkpoint blockade will likely offer a new strategy into combination therapies involving ECM remodeling and immune surveillance in advanced PDAC.

### 2.2. Regulatory T Cells (Tregs)

Tregs are characterized by expression of both CD4 and forkhead box P3 (FoxP3). Tregs are important to maintain immunological self-tolerance and have the ability to obstruct antitumor responses in several cancer types. High levels of Tregs are associated with immunosuppression and poor prognosis [35,36]. Infiltration of immunosuppressive cells such as Tregs and myeloid-derived suppressor cells (MDSCs) with procancerous signals is considered a factor contributing to the alteration of pro-inflammatory environment to tolerogenic immune surroundings [37]. Pancreatic cancer cells and PSCs produce Treg cell attractants CXCL10, CCL3, CCL4, CCL5, and vascular endothelial growth factor (VEGF) and interact with Treg cell-surface receptors CXCR3, CCR5, and neuropilin-1 to promote Treg cell migration and infiltration [38,39,40,41,42].

Activated Tregs create immunosuppressive circumstances via multiple mechanisms. For example, Tregs inhibit Teff activation and proliferation through IL-2 activation [43]. Tregs express inhibitory markers including CTLA-4 and inducible T cell costimulatory (ICOS) to accelerate apoptosis in Teff. Suppressive cytokines such as IL-10, IL-35, and transforming growth factor-β (TGF-β), are secreted by Tregs to diminish immune surveillance [44]. Importantly, pancreatic cancer mouse models have demonstrated that targeting CTLA4 in Tregs increases the infiltration of CD4+ T cells recruited to the tumor, suggesting that targeting Tregs is important for PDAC immunotherapy [45].

### 2.3. Myeloid Derived Suppressor Cells (MDSCs)

MDSCs are composed of heterogeneous immune cells with a phenotype of CD33^+^/HLA-DR^−/low^. MDSCs are associated with tumor progression, angiogenesis, senescence evasion, tumor metastasis, and chemoresistance. Their functions are repressive for T cell immunity and angiogenic to promote tumorigenesis [46,47]. Cytokines such as colony-stimulating factors are the main molecules stimulating generation of MDSCs from progenitor cells in bone marrow [48]. In mouse models, oncogenic Kras-induced GM-CSF production promotes MDSCs formation in the tumor microenvironment [49]. Besides, MDSCs are activated by several signals, including IL-4, IL-6, VEGF, and TGF-β [50,51,52,53].

It has been reported that mononuclear MDSCs have greater potential ability for suppressive control than polymorphonuclear MDSCs, and recruit FOXP3+ Treg cells by producing TGF-β, CCR5, and ARG-1 [54,55,56]. Hypoxic surroundings recruit MDSCs, activating the STAT3 pathway to secrete VEGF and basic fibroblast growth factor, which promote subsequent angiogenesis [57]. Depletion of MDSCs with Gr-1 antibody retarded the growth of pancreatic cancer cells and revealed a role for CD8 T cells in the disease [58]. 

### 2.4. Tumor-Associated Macrophages (TAMs)

Circulating monocytes are accumulated in the tumor microenvironment; they will proliferate and differentiate into different phenotypes of TAMs according to diverse stimulating signals. TAMs can be polarized into two different subtypes: (1) inflammatory M1-type macrophages, which are induced by IFN-γ, LPS, and IL-12, termed as “classical activation” and (2) precancerous M2-type macrophages, which are activated by IL-4 and IL-13, termed as “alternative activation” [59]. 

Besides cytokines, hypoxia and other signaling pathways such as IRF4, STAT6, MYC, PPARγ, and KLF4 have been reported to promote M2 polarization [60,61,62,63,64]. In general, M2 macrophages have an immunosuppressive phenotype and release cytokines, including IL-10, that induce a Th2 immune response [65]. In contrast, M1 macrophages are dominant and prone to tumor initiation and development in chronic inflammation [66]. After transformation to cancer, macrophages switch to the M2 phenotypes to invade and progress [67]. High infiltration of M2-type polarized TAMs presents poor prognosis in many cancers including pancreatic cancer. These evidences suggest that reversing the polarization of M2-type TAMs could provide a potential strategy in improving their immunosuppressive function in pancreatic cancer.

### 2.5. Dendritic Cells (DCs)

Antigen presentation via DCs is crucial to an effective antitumor T cell response. However, a paucity and disability of DCs is found in the pancreatic tumor microenvironment, with their location within the tumor being at the edges of cancer [68]. Similarly, the number of circulating DCs is relatively low in PDAC patients [69]. Studies indicate a positive correlation between high levels of blood DCs and better outcomes in patients with PDAC [70,71]. Notably, the co-inhibitory molecule indoleamine 2,3-dioxygenase (IDO), is increasingly expressed in the DCs of PDAC, which suppresses the T cell responses and promotes immune tolerance [72]. These data suggest that the tolerogenic functions of IDO can be targeted for treating pancreatic cancer, via modulation of DC function.

## 3. Immunotherapy in Pancreatic Cancer Treatment

The tumor microenvironment (TME) is involved in cancer biology in a more sophisticated manner in PDAC [73,74]. Evidences indicated that PDAC patients with both highest neoantigen number and the most abundant CD8+ T cell infiltrates have the longest survival time [75]. However, the activation signature of T cell is suppressed in genomic profiles of PDAC patients [76]. Immune cell and stromal signature integrating genomic and immunophenotypic classification of PDAC develops in recent studies [77,78]. The “immune rich” subtypes with rich in T and B cells, lower in FOXP3+ Tregs, and reduced CDKN2A and PIK3CA mutation rate, possess better outcome compared with “immune escape” and “immune exhausted” subtypes [78]. These signatures provide a new way to approach the immune response in PDAC patients with immunotherapy.

### 3.1. Checkpoint Inhibitors

Clinical trials of anti-PD-1/PD-L1 (BMS-936559, pembrolizumab, nivolumab, and atezolizumab) and anti-CTLA-4 (ipilimumab) monotherapies show no effects in unselected patients with advanced, pretreated, and progressive PDAC [18,19,79,80,81]. Only small groups of patients with mismatch repair (MMR) deficiency, which results in higher rates of somatic mutations and increased neoantigen production, showed higher clinical benefit with immune checkpoint inhibitors, despite the rare incidence rates in PDAC (less than 2%) [82,83,84,85]. Combination therapy is considered an alternative strategy to improve the clinical response via modulation of immune cell infiltration to the tumor region. These reports indicate that combination therapies display an advantage for the treatment of pancreatic cancer patients (Table 1). For example, acalabrutinib, a bruton’s tyrosine kinase inhibitor, which were used in lymphoma suppressed MDSCs and TAMs and promoted therapeutic potency after combining with checkpoint inhibitors [86,87]. CXCR4 antagonist (BL-8040) is a robust mobilizer of immune cells to peripheral blood and is effective at inducing direct tumor cell death. Additional findings have suggested that CXCR4 antagonists may be effective at increasing the infiltration of antitumor T cells into the tumor region. Therefore, combining BL-8040 with a checkpoint blockade is predicted to increase the responsiveness of PDAC patients to immunotherapy [88]. The efficacy of checkpoint inhibitors can also be improved in combination with dendritic cell-based therapy in pancreatic cancer via blockade of PD-L1 on dendritic cells, which induces a Th-1 immune profile and reduces the release of Th-2 cytokines [89]. Gemcitabine, the standard chemotherapy for pancreatic cancer treatment, increases the Teff:Treg ratio and the activity of Teff. Nab-paclitaxel and gemcitabine may also cooperate well with Nivolumab in EMT remodeling [90,91,92,93]. Furthermore, low-dose cyclophosphamide improved the antitumor immune responses when used in early cancer vaccine trials [94]. Daily and metronomic administration of cyclophosphamide effectively downregulated the number and function of Tregs in other cancers types [95,96]. In addition, an antivascular effect was also reported with low-dose cyclophosphamide in prostate cancer [97]. Although clinical trials of anti-CTLA-4 and anti-PD-1/PD-L1 therapies were well conducted, other checkpoint inhibitors including LAG-3, TIM-3, and A2AR have been applied in ongoing clinical trials [98].

### 3.2. Vaccinations 

Immunotherapy with vaccination aims to arouse passive immune responses via administering tumor-specific antigens and expressed epitopes for CD8+ and CD4+ T cells. These include whole-cell vaccines, DC vaccines, DNA vaccines, and peptide vaccines, which are all listed in Table 2. 

#### 3.2.1. GVAX 

GVAX is composed of tumor cells genetically modified to allogeneic cell lines that secrete the immune stimulatory cytokine, granulocyte-macrophage colony-stimulating factor (GM-CSF), and are then irradiated to prevent further cell division. GVAX stimulates stem cells to produce granulocytes and monocytes and promotes cytolytic activity against tumor cells [99]. In a pilot study, neoadjuvant and adjuvant GVAX was administered with or without cyclophosphamide in resected PDAC. Post-GVAX triggers the PD-1–PD-L1 axis to helpfully provide better candidates than vaccine-naive patients for immune checkpoints and other immunomodulatory therapies [100]. Notably, vaccine cells and their combination with 5-FU-based chemoradiation improved the clinical outcomes in resected PDAC patients (24.8 months, 95% CI, 21.2–31.6) [101,102]. 

#### 3.2.2. CRS-207 

CRS-207 is composed of live-attenuated, double-deleted *Listeria monocytogenes* expressing human mesothelin. CRS-207 has the ability to trigger both innate and mesothelin-specific CD8+ T cell induction adaptive immune responses to enhance GVAX-mediated antitumor effects in metastatic PDAC, compared to GVAX alone [103,104]. However, a disappointing result was noted in the phase IIb study, which showed no overall survival advantage in combination with cyclophosphamide (CY)/GVAX + CRS-207 in patients with previously treated metastatic PDAC, compared to chemotherapy [105]. 

#### 3.2.3. Algenpantucel-L 

Algenpantucel-L is composed of two human allogenic irradiated cancer lines genetically modified to express murine alpha-1,3-galactosyl transferase (αGT), which leads to synthesis of α-galactosyl (αGal) residues on their cell surface. Vaccine tumor cells mediate hyperacute rejection and phagocytosis to reinforce the anticancer immune response via cells with αGal expression [106]. A phase II adjuvant study with algenpantucel-L addition to standard adjuvant chemoradiotherapy reported that 12-month disease-free survival was 62% and that 12-month overall survival was 86% [107]. However, phase III randomized trials showed no overall survival difference between chemoradiotherapy with or without algenpantucel-L (30.4 months in control vs. 27.3 months in the treatment group) [108]. 

#### 3.2.4. *Kras* Vaccines 

It is well known that *Kras* point mutation triggers PanIN formation, which consequently progresses to PDAC development [109]. *Kras* mutation, mainly in codon 12, is commonly found in more than 90% of PDAC patients. Clarifying the detailed mechanisms may provide a useful strategy for immunotherapeutic treatments. It is known that mutant Kras peptides are processed and presented as foreign antigens by both MHC class I or II molecules [110,111], which become accessible to cytotoxic T cells. The products of mutant Kras antigens are expressed distinctively in tumor tissues compared to normal tissues. Vaccine peptides were custom synthesized to the corresponding mutation and injected to the patient to induce mutation-specific immune responses [112]. Besides, using nonpathogenic yeast as a vehicle to present mutated Kras peptide to DCs is another strategy for activating the immune system [113]. Unfortunately, the initial use of Kras vaccine in patients induced a transient T cell response but provided no clinical benefits [114]. However, combination with GM-CSF in adjuvant treatment induced a 58% response rate and longer median survival of responders than nonresponders (148 days vs. 61 days, respectively), and the survival of resectable patients was better than that of unresectable patients in a phase I/II trial [115]. Previous studies with long-term follow-up demonstrated 17 of 20 (85%) immune responders with a median survival of 28 months. The 10-year survival was 20% (4/20 evaluable) versus 0% in a comparable nonvaccinated cohort [116,117]. Another study of resectable pancreatic cancers with Kras mutation showed that concurrent use of Kras vaccine and GM-CSF resulted in 25% (9/24) immune responders. Median recurrence-free survival time was 8.6 months and median OS was 20.3 months [118].

#### 3.2.5. Telomerase Vaccine 

Telomerase is overexpressed in 85 to 90% of pancreas cancer cases, and immunogenic telomerase peptides have been defined as a target of anticancer drugs. Telomeres protect the end of the chromosome from DNA damage or from fusion with neighboring chromosomes. Telomerase is active in normal stem cells and in most cancer cells, but is nearly absent from most somatic cells. GV1001, a 16-aa hTERT peptide, belongs to cell-penetrating peptides facilitating hTERT-specific T cell response-mediated MHC activation. Notably, a telomerase peptide vaccine could interact with a variety of HLA-class II molecules including HLA-DR, -DP, and -DQ loci, thereby eliciting T helper (Th) responses commonly found in vaccinated patients [119]. Evidences indicate that higher immune responses are positively correlated with better survival [120]. In a phase I/II trial of unresectable PDAC, different dosages of GV1001 combined with GM-CSF were evaluated for immune response and survival in nonresectable PDAC patients. Median survival for the intermediate dose-group was significantly longer than that for the low- (*p* = 0.006) and high-dose groups (*p* = 0.05). Two phase III studies were conducted using a combination of chemotherapy agents, but showed no survival benefits in patients with metastatic PDAC. 

#### 3.2.6. Anti-Gastrin Vaccine 

Gastrin is expressed in the developing fetal pancreas; however, gastrin expression disappears in the embryo and is re-expressed in PanINs [121]. A gastrin-stimulating growth-mediated autocrine mechanism is commonly observed in pancreatic cancer [122]. Cholecystokinin (CCK) and gastrin are known to function in directly activating PSCs to further induce pancreatic fibrogenesis [123]. Antigastrin-17 vaccine (G17DT) is well known as a gastrin immunogen with a diphtheria toxoid (DT) carrier protein and plays an important role in regulating the tumor microenvironment. Two randomized clinical trials showed improved survival in responders compared to the placebo [124,125]. However, G17DT combined administration with gemcitabine showed inferior survival compared to gemcitabine plus placebo [126]. 

#### 3.2.7. Anti-VEGFR Vaccine 

Vascular endothelial growth factor receptor 2 (VEGFR2) is well characterized as a crucial regulator in driving tumor angiogenesis. VEGFR2 is a target for Treg recognition; therefore, VEGFR2+ Tregs demonstrate highly immunosuppressive activity in the tumor microenvironment [127]. In addition, VEGF inhibits the maturation of DCs and increases immune tolerance [128,129,130]. A phase I study combining VEGFR2-169 with gemcitabine in advanced PDAC demonstrated a 67% disease control rate and 8.7 months of median overall survival time [131].

## 4. Other Immunotherapies and Targeted Therapies Modulating the Tumor Microenvironment 

Stromal heterogeneity of numerous immune cells and components is involved in future therapeutic targets. Several therapies against stroma were investigated in clinical trials and are listed in Table 3. All-trans retinoic acid (ATRA) (NCT03307148) regulates the immune response via eradicating monocytic MDSCs, diminishing the suppressive capacity of granulocytic MDSCs in sarcomas [132], enhancing CD8+ T cell infiltration around cancer cells, and activating PSCs mediated by CXCL12 from PSCs in PDAC [133,134]. Paricalcitol (NCT02030860), a less calcemic analog of 1α,25(OH)_2_D_3_ and 19-nor-1α,25(OH)_2_D_2_, upregulates the expressions of p21 and p27 in vitro and in vivo leading to G0/G1 arrest in PDAC [135]*.* Paricalcitol also blocks high levels of vitamin D receptors on stellate cells and inactivates stroma production in PDAC [136]. Defactinib (NCT0254 6531), a second-generation inhibitor of focal adhesion kinase (FAK) and proline-rich tyrosine kinase-2, inhibits tumor cell survival and promotes anoikis. FAK signaling mediates the physical attachment of cells to ECM and promotes formation of a fibrotic and proinflammatory tumor microenvironment. Inhibition of FAK signaling leads to significant reduction in pancreatic tumor growth in animal models. FAK inhibitors displayed markedly reduced tumor fibrosis, decreased immunosuppressive MDSCs, and restored unresponsive Kras (G12D)/Trp53 null/Pdx1-cre (KPC) in mouse models of PDAC sensitive to PD-1 blockade [137,138]. Pexidartinib (NCT02777710), an inhibitor of M-CSF-receptor (M-CSFR) and c-kit tyrosine kinase, decreases CD206^+^ F4/80^+^ TAM (M2-like TAMs) number and blood vessel density, but improves the CD8+ T cell/Treg-ratio in malignant mesothelioma [139]. CXCR4 is a receptor specific for CXCL12 (also called stromal-derived-factor-1), a potent molecule with chemotactic activity for lymphocytes. Multiple targeted therapies including olaptesed and plerixafor (NCT03168139, NCT03277209, NCT02179970), target CXCR4/CXCL12 interaction, leading to modulation of the immune microenvironment. Olaptesed increases lymphocyte infiltration into solid tumor–stroma spheroids of different cancer cell lines, thereby synergizing with the anti-PD-1 checkpoint blockade [140]. Plerixafor may hinder the survival, growth, and migration of CXCR4-expressing cancer cells and inhibit endothelial progenitor cells to reduce tumor vasculogenesis in ovarian and breast cancers [141,142]. In Ewing Tumors, plerixafor diminishes PDGFB expression and results in compromised tumor vasculature and apoptosis in vivo [143]. Hypoxia and anti-angiogenesis also obstruct the proliferation of pancreatic cancer cells, and similar agents including evofosfamide (NCT03098160), apatinib (NCT02726854), and ziv-aflibercept (NCT02159989) reduce the oxygen and nutrient supply to the tumor. In several cancers including PDAC, Lenalidomide (NCT01547260) exerts diverse antitumor effects through anti-angiogenesis and recruitment of tumor antigen-specific T cells, thus enhancing natural killer (NK) cell cytotoxicity [144,145,146]. In addition, lenalidomide promotes T cells and induces proliferation, cytokine production, and cytotoxic activity, with decreased TNF-α and IL-12 production [145,147]. Trabedersen (NCT00844064), a TGF-β antagonist, functions to overcome TGF-β2-mediated immunosuppression in pancreatic cancer cells. Trabedersen induces LAK (lymphokine activated killer) cell-mediated cytotoxicity in patients with pancreatic cancer, malignant melanoma, and colorectal carcinoma [148,149]. TGF-β inhibitors enhance immunosurveillance and reinforce immune recognition in the tumor microenvironment.

## 5. Perspectives and Conclusions

**Figure 1:**
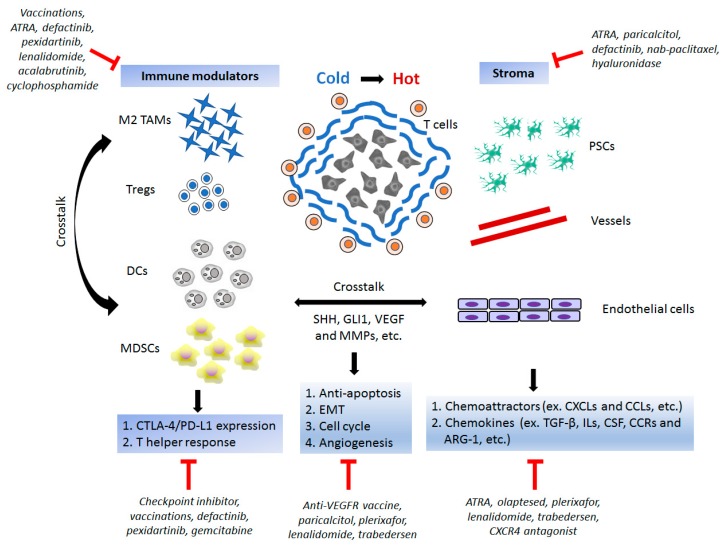
The response to immunotherapy in pancreatic ductal adenocarcinoma (PDAC) relies on destroying cancer cells as well as on breaking the stromal barrier and flaring up immune function. Immunotherapy targeting the PD-1/PD-L1 pathway and TME has disrupted the traditional method of cancer treatment and countered the immune-related adverse events in patients. In addition to direct cytotoxicity on cancer cells, it is important to dissect the immunosuppressive microenvironment with various cytokines and immune cells. Furthermore, recognition of mechanisms modulating the PD-1/PD-L1 pathway and TME will identify more potential therapeutic targets in the future.

Though the results of checkpoint inhibitors do not meet the expectations, PD-1/PD-L1 pathway is still considered vital to regulate antitumor activity in pancreatic cancer. The residual components including PSCs, Tregs, MDSCs, TAMs, and DCs in the tumor microenvironment play a decisive role in reversing immune dysfunction and modulating the effect of checkpoint inhibitors. Early phase clinical trials in PDAC showed that monotherapy with blockade of the PD-1/PD-L1 pathway did not attain satisfactory responses. However, the safety profiles and toxicity with anti-PD-1 monoclonal antibodies are acceptable, leading to increased interest in targeting PD-1/PD-L1 in PDAC. After combining PD-1/PD-L1 antibodies with agents altering TME, such as chemotherapies, targeted therapies, and vaccination, higher response rates are demonstrated in advanced and pretreated PDAC patients. Unlike other types of neoplasms, passive immunity is more crucial and acts as an “enhancer” for immunologically ignorant pancreatic cancer. Vaccination may turn the tumor microenvironment from a “cold” tumor to “hot” tumor and arouse immunosurveillance and immunoediting, after which the anti-PD-1 antibody boosts tumor cell destruction. Diverse combinations of vaccine therapies are undergoing clinical trials and are listed in Table 4. Multimodalities of treatment strategies will be needed for overcoming the fibrotic stroma, suppressive immune cells, and malignant cancer cells. Early phase clinical trials conducted with oncolytic viruses, TGFβ inhibitors, and SMO inhibitors in advanced PDAC patients have shown preliminary promising results [149,150,151,152,153,154]. Novel strategies targeting the immune check points and stroma-associated therapies have also revealed impressive results in preclinical studies. For example, blockade of the TGFβ pathway combined with nivolumab treatment provides immune restoration to enhance immune responses and promote tumor regression [155]. As TGFβ inhibitors are currently used in recurrent/refractory high-grade glioma [156], triple combinations with checkpoint inhibitors and vaccination may be a potential and novel therapeutic strategy in PDAC patients. On the other hand, these combinations may conquer the resistance mechanisms to the PD-1/PD-L1 pathway blockade (Figure 1). 

## Figures and Tables

**Table 1:** Summary of clinical trials on checkpoint inhibitor monotherapies or combination therapy in pancreatic cancer.

Molecules	Regimen	Phase	*n*	Patient Population	Results	ORR (Responder/*n*)	Survival	NCT Number
BMS-936559	Monotherapy	I	14	Advanced, pretreated	Negative	0%		NCT00729664
Ipilimumab	Monotherapy	II	27	Advanced, pretreated	Negative	0%		NCT00112580
	Combination with GVAX	Ib	30	Advanced, pretreated	Positive	0%	OS: 5.7 m 1 y OS: 27%	NCT00836407
Atezolizumab	Monotherapy	I	1	Advanced, pretreated	Negative	0%	PFS: 12.2 m	NCT02302807
Pembrolizumab	Monotherapy	I	1	Advanced, pretreated	Negative	0%		NCT02331251
	Monotherapy	II	4	Advanced, pretreated	Positive	50% (2/4)		NCT01876511
	Combination with acalabrutinib	II	28	Advanced, pretreated	Positive	7.1% (1/14)		NCT02362048
	Combination with BL-8040	II	37	Advanced, pretreated	Positive	3.4% (1/29)	OS: 3.4 m (all) 7.5 m (2 L)	NCT02826486
Nivolumab	Combination with nab-paclitaxel +/− gemcitabine	I	6	Advanced	Positive	18.2% (2/11) (−gem) 50% (3/6) (+gem)		NCT02309177
	Combination with cyclophosphamide/GVAX +/− CRS-207	II	90	Advanced	Positive		OS: 3.9 m (−CRS) 6.1 m (+CRS)	NCT01417000
	Combination with MoDC		7	Advanced, pretreated	Positive	28.6% (2/7)	OS: 8 m and 16 m (responder)	Investigator-initiated trail

The legends of abbreviations: *n*: patient numbers, ORR: overall response rate, OS: overall survival, PFS: progression-free survival, m: months, 2 L: second-line, gem: gemcitabine, and MoDC: monocyte derived dendritic cells.

**Table 2:** Summary of mechanisms and trials on vaccination treatments that modulate the tumor microenvironment.

Agents	Mechanisms on Immune System	Clinical Trials
GVAX	Increased tumor antigen recognition by the immune system through presentation by dendritic cells, including mesothelin Induces intratumoral tertiary lymphoid structures and TILs Upregulates PD-L1 membranous expression on cancer cells Delayed-type hypersensitivity	Novel Allogeneic Granulocyte-macrophage colony-stimulating Factor-secreting Tumor Vaccine for Pancreatic Cancer: A Phase I Trial of Safety and Immune activation, NTC03122106. Vaccine Therapy Combined With Adjuvant Chemoradiotherapy in Treating Patients With Resected Stage I or Stage II Adenocarcinoma (Cancer) of the Pancreas, NCT00084383. Safety and Efficacy of Combination Listeria/GVAX Immunotherapy in Pancreatic Cancer, NCT01417000.
CRS-207	Stimulates potent innate and mesothelin-specific adaptive immunity and “boosts” the immune response initiated by GVAX Induces T cells to leave the periphery and enter tissues	Safety and Efficacy of Combination Listeria/GVAX Immunotherapy in Pancreatic Cancer, NCT01417000. Safety and Efficacy of Combination Listeria/GVAX Pancreas Vaccine in the Pancreatic Cancer Setting (ECLIPSE), NCT02004262.
Algenpantucel-L	Mediates hyperacute rejection Anti-αGal antibodies bind to αGal epitopes causing complement- and antibody-dependent cell-mediated destruction of transplanted allografts	Immunotherapy Study for Surgically Resected Pancreatic Cancer, NCT01072981. Vaccine Study for Surgically Resected Pancreatic Cancer, NCT00569387. Vaccine Treatment for Surgically Resected Pancreatic Cancer, NCT00255827.
KRAS peptide (including GI4000 etc.)	Presented as a foreign antigen by MHC class I and II to CD4+ and CD8+ T cells, and induces cytotoxic effects Using nonpathogenic yeast as a vehicle to carry antigens and present them to DCs Lowers the expression of FoxP3 cells Increases the ratio of CD4+CD25+ activated T cells to Tregs Increase production of Th1-related cytokines and IL-6 Delayed-type hypersensitivity	Safety and Efficacy of the Therapeutic Vaccine GI-4000 in Combination With Gemcitabine Versus Placebo for the Treatment of Non-metastatic, Post-resection Pancreas Cancer, NCT00300950. Vaccine Therapy in Treating Patients With Colon, Pancreatic, or Lung Cancer, NCT00019006. Vaccine Therapy and Biological Therapy in Treating Patients With Advanced Cancer, NCT00019084. Vaccine Therapy Plus Biological Therapy in Treating Adults With Metastatic Solid Tumors, NCT00019331. Vaccine Therapy Plus QS21 in Treating Patients With Advanced Pancreatic or Colorectal Cancer, NCT00006387.
Telomerase peptide (GV1001)	Facilitates the transport of molecular cargo across the plasma membrane Binds to MHC, activating hTERT-specific T cell responses Integrates both T helper and CTL responses Delayed-type hypersensitivity	Gemcitabine and Capecitabine With or Without Vaccine Therapy in Treating Patients With Locally Advanced or Metastatic Pancreatic Cancer, NCT00425360. Immunochemoradiotherapy in Patients With Pancreatic Cancer, NCT01342224. A Feasibility and Safety Study of Vaccination With Poly-ICLC and Peptide-pulsed Dendritic Cells in Patients With Metastatic, Locally Advanced, Unresectable, or Recurrent Pancreatic Adenocarcinoma, NCT01410968.
Antigastrin-17 vaccine (G17DT)	Gastrin is a driver of pancreatic cancer that stimulates growth through a markedly overexpressed CCK receptor Reverse intense desmoplastic reaction of pancreatic cancer via modulating pancreatic stellate cells Arrests the progression of PanINs	An Open Label Study to Evaluate G17DT Compared to Gemcitabine, NCT03200821. An Open, Single-center Study to Determine the Antibody Response to Gastrimmune and Its Safety and Tolerability in Patients With Advanced Pancreatic Carcinoma, NCT02098291. Safety and Efficacy of G17DT Immunogen Combined With Gemcitabine vs. Gemcitabine in the Treatment of Advanced Pancreatic Carcinoma, NCT00044031. Sequential Trial of G17DT for the Treatment of Advanced Pancreatic Cancer, NCT02118077. Single Centre Study to Determine the Antibody Response to G17DT in Patients With Advanced Pancreatic Cancer, NCT02098239
Anti-VEGFR vaccine (VEGFR2-169)	Reduces the number and function of Tregs Inhibits the infiltration of other suppressive immune cells (MDSCs, macrophages) Activation of CD4+ T cells Increases the mature dendritic cell fraction Changes the intratumoral cytokine levels, specifically those of IL-1β, IL-6, and CXCL1 Reduces the production and expression of interleukin-10 and TGF-β in TME	Gemcitabine With Antiangiogenic Peptide Vaccine Therapy in Patients With Pancreatic Cancer, NCT00622622 Antiangiogenic Peptide Vaccine Therapy With Gemcitabine in Treating Patient With Pancreatic Cancer (Phase1/2), NCT00655785

**Table 3:** Summary of mechanisms in targeting the tumor microenvironment (TME).

Agents	Mechanisms	References
ATRA	Eradicates monocytic MDSCs and diminishes the suppressive capacity of granulocytic MDSCs Enhances the CD8+ T cell infiltrate around cancer cells Activate PSCs mediated by CXCL12 from PSCs	[132,133,134]
Paricalcitol	Inhibits CDK2, CDK4, Cyclin D1, Cyclin E, and Cyclin A Upregulates the expressions of p21 and p27 in vitro and in vivo Blocks high levels of vitamin D receptors on stellate cells and inactivates stromal production	[135,136]
Defactinib	Inhibits physical attachment of cells to the ECM Inhibit tumor fibrosis and proinflammatory tumor microenvironment Decreases immunosuppressive MDSCs Render the previously unresponsive KPC mouse models sensitive to PD-1 blockade	[137,138]
Pexidartinib	Decreases CD206^+^ F4/80^+^ TAM (M2-like TAMs) numbers and blood vessel density Improves the CD8+ T cell/Treg-ratio	[139]
Olaptesed	Targets CXCL12 Increases lymphocyte infiltration into solid tumor–stroma spheroids, thereby synergizing with the anti-PD-1 checkpoint blockade Lowers the monocyte-to-lymphocyte ratio	[140]
Plerixafor	Targets CXCR4 Hinders the survival, growth, and migration of CXCR4-expressing cancer cells Inhibits endothelial progenitor cells to reduce tumor vasculogenesis Diminishes PDGFB expression resulting in compromised tumor vasculature and apoptosis in vivo	[141,142,143]
Lenalidomide	Anti-angiogenesis Expands tumor antigen-specific T cells and enhances natural killer (NK) cell cytotoxicity Promotes T cells inducing proliferation, cytokine production, and cytotoxic activity Decreases TNF-α and interleukin-12 production	[144,145,147]
Trabedersen	Reverses TGF-β2-mediated immunosuppression of pancreatic cancer cells Increases LAK (lymphokine activated killer) cell-mediated cytotoxicity to pancreatic cancer cells Prevents angiogenesis	[148,149]

**Table 4:** Summary of ongoing clinical trials on vaccination.

Agents	Ongoing Clinical Trials
GVAX	Neoadjuvant/Adjuvant GVAX Pancreas Vaccine (With CY) With or Without Nivolumab Trial for Surgically Resectable Pancreatic Cancer, NCT02451982, status: recruiting Study With CY, Pembrolizumab, GVAX, and SBRT in Patients With Locally Advanced Pancreatic Cancer, NCT02648282, status: recruiting Study of CRS-207, Nivolumab, and Ipilimumab With or Without GVAX Pancreas Vaccine (With Cy) in Patients With Pancreatic Cancer, NCT03190265, status: recruiting Phase 2 GVAX Pancreas Vaccine (With CY) in Combination With Nivolumab and SBRT for Patients With Borderline Resectable Pancreatic Cancer, NCT03161379, status: recruiting Pancreatic Tumor Cell Vaccine (GVAX), Low Dose Cyclophosphamide, Fractionated Stereotactic Body Radiation Therapy (SBRT), and FOLFIRINOX Chemotherapy in Patients With Resected Adenocarcinoma of the Pancreas, NCT01595321, status: active, not recruiting Pilot Study With CY, Pembrolizumab, GVAX, and IMC-CS4 (LY3022855) in Patients With Borderline Resectable Adenocarcinoma of the Pancreas, NCT03153410, status: recruiting Vaccine Therapy With or Without Cyclophosphamide in Treating Patients Undergoing Chemotherapy and Radiation Therapy for Stage I or Stage II Pancreatic Cancer That Can Be Removed by Surgery, NCT00727441, status: active, not recruiting
CRS-207	Study of CRS-207, Nivolumab, and Ipilimumab With or Without GVAX Pancreas Vaccine (With Cy) in Patients With Pancreatic Cancer, NCT03190265, status: recruiting Epacadostat, Pembrolizumab, and CRS-207, With or Without CY/GVAX Pancreas in Patients With Metastatic Pancreas Cancer, NCT03006302, status: recruiting GVAX Pancreas Vaccine (With CY) and CRS-207 With or Without Nivolumab, NCT02243371, status: active, not recruiting Study of Safety and Tolerability of Intravenous CRS-207 in Adults With Selected Advanced Solid Tumors Who Have Failed or Who Are Not Candidates for Standard Treatment, NCT00585845, status: terminated
Algenpantucel-L	Immunotherapy and SBRT Study in Borderline Resectable Pancreatic Cancer, NCT02405585, status: terminated Low Dose Vaccine Study for Surgically Resected Pancreatic Cancer, NCT00614601, status: terminated Long Term Follow-Up Study for Subjects Previously Treated With Algenpantucel-L (HyperAcute-Pancreas) Immunotherapy, NCT03165188, status: recruiting
KRAS peptide	QUILT-3.070: Pancreatic Cancer Vaccine: Subjects With Pancreatic Cancer Who Have Progressed on or After Standard-of-care Therapy, NCT03387098, status: recruiting QUILT-3.060: NANT Pancreatic Cancer Vaccine: Molecularly Informed Integrated Immunotherapy in Subjects With Pancreatic Cancer Who Have Progressed on or After Standard-of-care Therapy, NCT03329248, status: active, not recruiting QUILT-3.080: NANT Pancreatic Cancer Vaccine, NCT03586869, status: recruiting QUILT-3.039: NANT Pancreatic Cancer Vaccine: Combination Immunotherapy in Subjects With Pancreatic Cancer Who Have Progressed on or After Standard-of-care Therapy, NCT03136406, status: active, not recruiting QUILT-3.088: NANT Pancreatic Cancer Vaccine, NCT03563144, status: not yet recruiting Vaccine Therapy in Treating Patients With Pancreatic Cancer That Has Been Removed by Surgery, NCT00389610, status: active, not recruiting
Telomerase peptide (GV1001)	hTERT Immunotherapy Alone or in Combination With IL-12 DNA Followed by Electroporation in Adults With Solid Tumors at High Risk of Relapse, NCT02960594, status: active, not recruiting

## References

[1] Ryan D.P., Hong T.S., Bardeesy N. (2014). Pancreatic adenocarcinoma. N. Engl. J. Med..

[2] Louvet C., Labianca R., Hammel P., Lledo G., Zampino M.G., Andre T., Zaniboni A., Ducreux M., Aitini E., Taieb J. (2005). Gemcitabine in combination with oxaliplatin compared with gemcitabine alone in locally advanced or metastatic pancreatic cancer: Results of a GERCOR and GISCAD phase III trial. J. Clin. Oncol..

[3] Burris H.A., Moore M.J., Andersen J., Green M.R., Rothenberg M.L., Modiano M.R., Cripps M.C., Portenoy R.K., Storniolo A.M., Tarassoff P. (1997). Improvements in survival and clinical benefit with gemcitabine as first-line therapy for patients with advanced pancreas cancer: A randomized trial. J. Clin. Oncol..

[4] Neoptolemos J.P., Stocken D.D., Bassi C., Ghaneh P., Cunningham D., Goldstein D., Padbury R., Moore M.J., Gallinger S., Mariette C. (2010). Adjuvant chemotherapy with fluorouracil plus folinic acid vs. gemcitabine following pancreatic cancer resection: A randomized controlled trial. JAMA.

[5] Uesaka K., Boku N., Fukutomi A., Okamura Y., Konishi M., Matsumoto I., Kaneoka Y., Shimizu Y., Nakamori S., Sakamoto H. (2016). Adjuvant chemotherapy of S-1 versus gemcitabine for resected pancreatic cancer: A phase 3, open-label, randomised, non-inferiority trial (JASPAC 01). Lancet (Lond. Engl.).

[6] Conroy T., Desseigne F., Ychou M., Bouche O., Guimbaud R., Becouarn Y., Adenis A., Raoul J.L., Gourgou-Bourgade S., de la Fouchardiere C. (2011). FOLFIRINOX versus gemcitabine for metastatic pancreatic cancer. N. Engl. J. Med..

[7] Goldstein D., El-Maraghi R.H., Hammel P., Heinemann V., Kunzmann V., Sastre J., Scheithauer W., Siena S., Tabernero J., Teixeira L. (2015). nab-Paclitaxel plus gemcitabine for metastatic pancreatic cancer: Long-term survival from a phase III trial. J. Natl. Cancer Inst..

[8] Dunn G.P., Old L.J., Schreiber R.D. (2004). The immunobiology of cancer immunosurveillance and immunoediting. Immunity.

[9] Korman A.J., Peggs K.S., Allison J.P. (2006). Checkpoint blockade in cancer immunotherapy. Adv. Immunol..

[10] Ribas A. (2008). Overcoming immunologic tolerance to melanoma: Targeting CTLA-4 with tremelimumab (CP-675,206). Oncologist.

[11] Weber J. (2008). Overcoming immunologic tolerance to melanoma: Targeting CTLA-4 with ipilimumab (MDX-010). Oncologist.

[12] Freeman G.J., Long A.J., Iwai Y., Bourque K., Chernova T., Nishimura H., Fitz L.J., Malenkovich N., Okazaki T., Byrne M.C. (2000). Engagement of the PD-1 immunoinhibitory receptor by a novel B7 family member leads to negative regulation of lymphocyte activation. J. Exp. Med..

[13] Riley J.L. (2009). PD-1 signaling in primary T cells. Immunol. Rev..

[14] Dong H., Strome S.E., Salomao D.R., Tamura H., Hirano F., Flies D.B., Roche P.C., Lu J., Zhu G., Tamada K. (2002). Tumor-associated B7-H1 promotes T-cell apoptosis: A potential mechanism of immune evasion. Nat. Med..

[15] Boussiotis V.A. (2016). Molecular and Biochemical Aspects of the PD-1 Checkpoint Pathway. N. Engl. J. Med..

[16] Keir M.E., Butte M.J., Freeman G.J., Sharpe A.H. (2008). PD-1 and its ligands in tolerance and immunity. Annu. Rev. Immunol..

[17] Tumeh P.C., Harview C.L., Yearley J.H., Shintaku I.P., Taylor E.J., Robert L., Chmielowski B., Spasic M., Henry G., Ciobanu V. (2014). PD-1 blockade induces responses by inhibiting adaptive immune resistance. Nature.

[18] Patnaik A., Kang S.P., Rasco D., Papadopoulos K.P., Elassaiss-Schaap J., Beeram M., Drengler R., Chen C., Smith L., Espino G. (2015). Phase I Study of Pembrolizumab (MK-3475; Anti-PD-1 Monoclonal Antibody) in Patients with Advanced Solid Tumors. Clin. Cancer Res..

[19] Brahmer J.R., Tykodi S.S., Chow L.Q., Hwu W.J., Topalian S.L., Hwu P., Drake C.G., Camacho L.H., Kauh J., Odunsi K. (2012). Safety and activity of anti-PD-L1 antibody in patients with advanced cancer. N. Engl. J. Med..

[20] Topalian S.L., Hodi F.S., Brahmer J.R., Gettinger S.N., Smith D.C., McDermott D.F., Powderly J.D., Carvajal R.D., Sosman J.A., Atkins M.B. (2012). Safety, activity, and immune correlates of anti-PD-1 antibody in cancer. N. Engl. J. Med..

[21] Balar A.V., Weber J.S. (2017). PD-1 and PD-L1 antibodies in cancer: Current status and future directions. Cancer Immunol. Immunother. CII.

[22] Bhaw-Luximon A., Jhurry D. (2015). New avenues for improving pancreatic ductal adenocarcinoma (PDAC) treatment: Selective stroma depletion combined with nano drug delivery. Cancer Lett..

[23] Zhuan-Sun Y., Huang F., Feng M., Zhao X., Chen W., Zhu Z., Zhang S. (2017). Prognostic value of PD-l1 overexpression for pancreatic cancer: Evidence from a meta-analysis. OncoTargets Ther..

[24] Hegde P.S., Karanikas V., Evers S. (2016). The Where, the When, and the How of Immune Monitoring for Cancer Immunotherapies in the Era of Checkpoint Inhibition. Clin. Cancer Res..

[25] Apte M.V., Park S., Phillips P.A., Santucci N., Goldstein D., Kumar R.K., Ramm G.A., Buchler M., Friess H., McCarroll J.A. (2004). Desmoplastic reaction in pancreatic cancer: Role of pancreatic stellate cells. Pancreas.

[26] Pandol S., Gukovskaya A., Edderkaoui M., Dawson D., Eibl G., Lugea A. (2012). Epidemiology, risk factors, and the promotion of pancreatic cancer: Role of the stellate cell. J. Gastroenterol. Hepatol..

[27] Spivak-Kroizman T.R., Hostetter G., Posner R., Aziz M., Hu C., Demeure M.J., Von Hoff D., Hingorani S.R., Palculict T.B., Izzo J. (2013). Hypoxia triggers hedgehog-mediated tumor-stromal interactions in pancreatic cancer. Cancer Res..

[28] Martinez-Bosch N., Fernandez-Barrena M.G., Moreno M., Ortiz-Zapater E., Munne-Collado J., Iglesias M., Andre S., Gabius H.J., Hwang R.F., Poirier F. (2014). Galectin-1 drives pancreatic carcinogenesis through stroma remodeling and Hedgehog signaling activation. Cancer Res..

[29] Yauch R.L., Gould S.E., Scales S.J., Tang T., Tian H., Ahn C.P., Marshall D., Fu L., Januario T., Kallop D. (2008). A paracrine requirement for hedgehog signalling in cancer. Nature.

[30] Tian H., Callahan C.A., DuPree K.J., Darbonne W.C., Ahn C.P., Scales S.J., de Sauvage F.J. (2009). Hedgehog signaling is restricted to the stromal compartment during pancreatic carcinogenesis. Proc. Natl. Acad. Sci. USA.

[31] Onishi H., Katano M. (2014). Hedgehog signaling pathway as a new therapeutic target in pancreatic cancer. World J. Gastroenterol..

[32] Hanna A., Shevde L.A. (2016). Hedgehog signaling: Modulation of cancer properies and tumor mircroenvironment. Mol. Cancer.

[33] Kawase T., Yasui Y., Nishina S., Hara Y., Yanatori I., Tomiyama Y., Nakashima Y., Yoshida K., Kishi F., Nakamura M. (2015). Fibroblast activation protein-alpha-expressing fibroblasts promote the progression of pancreatic ductal adenocarcinoma. BMC Gastroenterol..

[34] Ozdemir B.C., Pentcheva-Hoang T., Carstens J.L., Zheng X., Wu C.C., Simpson T.R., Laklai H., Sugimoto H., Kahlert C., Novitskiy S.V. (2014). Depletion of carcinoma-associated fibroblasts and fibrosis induces immunosuppression and accelerates pancreas cancer with reduced survival. Cancer Cell.

[35] Josefowicz S.Z., Lu L.F., Rudensky A.Y. (2012). Regulatory T cells: Mechanisms of differentiation and function. Annu. Rev. Immunol..

[36] Clark C.E., Hingorani S.R., Mick R., Combs C., Tuveson D.A., Vonderheide R.H. (2007). Dynamics of the immune reaction to pancreatic cancer from inception to invasion. Cancer Res..

[37] Zheng L., Xue J., Jaffee E.M., Habtezion A. (2013). Role of immune cells and immune-based therapies in pancreatitis and pancreatic ductal adenocarcinoma. Gastroenterology.

[38] Jang J.E., Hajdu C.H., Liot C., Miller G., Dustin M.L., Bar-Sagi D. (2017). Crosstalk between Regulatory T Cells and Tumor-Associated Dendritic Cells Negates Anti-tumor Immunity in Pancreatic Cancer. Cell Rep..

[39] Lunardi S., Lim S.Y., Muschel R.J., Brunner T.B. (2015). IP-10/CXCL10 attracts regulatory T cells: Implication for pancreatic cancer. Oncoimmunology.

[40] Tan M.C., Goedegebuure P.S., Belt B.A., Flaherty B., Sankpal N., Gillanders W.E., Eberlein T.J., Hsieh C.S., Linehan D.C. (2009). Disruption of CCR5-dependent homing of regulatory T cells inhibits tumor growth in a murine model of pancreatic cancer. J. Immunol..

[41] Hansen W., Hutzler M., Abel S., Alter C., Stockmann C., Kliche S., Albert J., Sparwasser T., Sakaguchi S., Westendorf A.M. (2012). Neuropilin 1 deficiency on CD4+Foxp3+ regulatory T cells impairs mouse melanoma growth. J. Exp. Med..

[42] Schlecker E., Stojanovic A., Eisen C., Quack C., Falk C.S., Umansky V., Cerwenka A. (2012). Tumor-infiltrating monocytic myeloid-derived suppressor cells mediate CCR5-dependent recruitment of regulatory T cells favoring tumor growth. J. Immunol..

[43] Pandiyan P., Zheng L., Ishihara S., Reed J., Lenardo M.J. (2007). CD4+CD25+Foxp3+ regulatory T cells induce cytokine deprivation-mediated apoptosis of effector CD4+ T cells. Nat. Immunol..

[44] Vignali D.A., Collison L.W., Workman C.J. (2008). How regulatory T cells work. Nat. Rev. Immunol..

[45] Bengsch F., Knoblock D.M., Liu A., McAllister F., Beatty G.L. (2017). CTLA-4/CD80 pathway regulates T cell infiltration into pancreatic cancer. Cancer Immunol. Immunother. CII.

[46] Di Mitri D., Toso A., Alimonti A. (2015). Tumor-infiltrating myeloid cells drive senescence evasion and chemoresistance in tumors. Oncoimmunology.

[47] Takeuchi S., Baghdadi M., Tsuchikawa T., Wada H., Nakamura T., Abe H., Nakanishi S., Usui Y., Higuchi K., Takahashi M. (2015). Chemotherapy-Derived Inflammatory Responses Accelerate the Formation of Immunosuppressive Myeloid Cells in the Tissue Microenvironment of Human Pancreatic Cancer. Cancer Res..

[48] Serafini P., Carbley R., Noonan K.A., Tan G., Bronte V., Borrello I. (2004). High-dose granulocyte-macrophage colony-stimulating factor-producing vaccines impair the immune response through the recruitment of myeloid suppressor cells. Cancer Res..

[49] Pylayeva-Gupta Y., Lee K.E., Hajdu C.H., Miller G., Bar-Sagi D. (2012). Oncogenic Kras-induced GM-CSF production promotes the development of pancreatic neoplasia. Cancer Cell.

[50] Fang Z., Li J., Yu X., Zhang D., Ren G., Shi B., Wang C., Kosinska A.D., Wang S., Zhou X. (2015). Polarization of Monocytic Myeloid-Derived Suppressor Cells by Hepatitis B Surface Antigen Is Mediated via ERK/IL-6/STAT3 Signaling Feedback and Restrains the Activation of T Cells in Chronic Hepatitis B Virus Infection. J. Immunol..

[51] Lee C.R., Kwak Y., Yang T., Han J.H., Park S.H., Ye M.B., Lee W., Sim K.Y., Kang J.A., Kim Y.C. (2016). Myeloid-Derived Suppressor Cells Are Controlled by Regulatory T Cells via TGF-beta during Murine Colitis. Cell Rep..

[52] Bronte V., Serafini P., De Santo C., Marigo I., Tosello V., Mazzoni A., Segal D.M., Staib C., Lowel M., Sutter G. (2003). IL-4-induced arginase 1 suppresses alloreactive T cells in tumor-bearing mice. J. Immunol..

[53] Ko J.S., Rayman P., Ireland J., Swaidani S., Li G., Bunting K.D., Rini B., Finke J.H., Cohen P.A. (2010). Direct and differential suppression of myeloid-derived suppressor cell subsets by sunitinib is compartmentally constrained. Cancer Res..

[54] Movahedi K., Guilliams M., Van den Bossche J., Van den Bergh R., Gysemans C., Beschin A., De Baetselier P., Van Ginderachter J.A. (2008). Identification of discrete tumor-induced myeloid-derived suppressor cell subpopulations with distinct T cell-suppressive activity. Blood.

[55] Nakamura T., Nakao T., Ashihara E., Yoshimura N. (2016). Myeloid-derived Suppressor Cells Recruit CD4(+)/Foxp3(+) Regulatory T Cells in a Murine Cardiac Allograft. Transplant. Proc..

[56] Kang X., Zhang X., Liu Z., Xu H., Wang T., He L., Zhao A. (2016). Granulocytic myeloid-derived suppressor cells maintain feto-maternal tolerance by inducing Foxp3 expression in CD4+CD25-T cells by activation of the TGF-beta/beta-catenin pathway. Mol. Hum. Reprod..

[57] Wang J., Su X., Yang L., Qiao F., Fang Y., Yu L., Yang Q., Wang Y., Yin Y., Chen R. (2016). The influence of myeloid-derived suppressor cells on angiogenesis and tumor growth after cancer surgery. Int. J. Cancer.

[58] Stromnes I.M., Brockenbrough J.S., Izeradjene K., Carlson M.A., Cuevas C., Simmons R.M., Greenberg P.D., Hingorani S.R. (2014). Targeted depletion of an MDSC subset unmasks pancreatic ductal adenocarcinoma to adaptive immunity. Gut.

[59] Stein M., Keshav S., Harris N., Gordon S. (1992). Interleukin 4 potently enhances murine macrophage mannose receptor activity: A marker of alternative immunologic macrophage activation. J. Exp. Med..

[60] Leblond M.M., Gerault A.N., Corroyer-Dulmont A., MacKenzie E.T., Petit E., Bernaudin M., Valable S. (2016). Hypoxia induces macrophage polarization and re-education toward an M2 phenotype in U87 and U251 glioblastoma models. Oncoimmunology.

[61] Colegio O.R. (2016). Lactic acid polarizes macrophages to a tumor-promoting state. Oncoimmunology.

[62] Rojas A., Delgado-Lopez F., Perez-Castro R., Gonzalez I., Romero J., Rojas I., Araya P., Anazco C., Morales E., Llanos J. (2016). HMGB1 enhances the protumoral activities of M2 macrophages by a RAGE-dependent mechanism. Tumour Biol..

[63] Biswas S.K., Allavena P., Mantovani A. (2013). Tumor-associated macrophages: Functional diversity, clinical significance, and open questions. Semin. Immunopathol..

[64] Mantovani A., Allavena P. (2015). The interaction of anticancer therapies with tumor-associated macrophages. J. Exp. Med..

[65] Mantovani A., Sica A. (2010). Macrophages, innate immunity and cancer: Balance, tolerance, and diversity. Curr. Opin. Immunol..

[66] Karin M., Greten F.R. (2005). NF-kappaB: Linking inflammation and immunity to cancer development and progression. Nat. Rev. Immunol..

[67] Ruffell B., Affara N.I., Coussens L.M. (2012). Differential macrophage programming in the tumor microenvironment. Trends Immunol..

[68] Dallal R.M., Christakos P., Lee K., Egawa S., Son Y.-I., Lotze M.T. (2002). Paucity of dendritic cells in pancreatic cancer. Surgery.

[69] Tjomsland V., Sandström P., Spångeus A., Messmer D., Emilsson J., Falkmer U., Falkmer S., Magnusson K.-E., Borch K., Larsson M. (2010). Pancreatic adenocarcinoma exerts systemic effects on the peripheral blood myeloid and plasmacytoid dendritic cells: An indicator of disease severity?. BMC Cancer.

[70] Hirooka S., Yanagimoto H., Satoi S., Yamamoto T., Toyokawa H., Yamaki S., Yui R., Inoue K., Michiura T., Kwon A.-H. (2011). The role of circulating dendritic cells in patients with unresectable pancreatic cancer. Anticancer Res..

[71] Yamamoto T., Yanagimoto H., Satoi S., Toyokawa H., Yamao J., Kim S., Terakawa N., Takahashi K., Kwon A.-H. (2012). Circulating myeloid dendritic cells as prognostic factors in patients with pancreatic cancer who have undergone surgical resection. J. Surg. Res..

[72] Munn D.H., Mellor A.L. (2016). IDO in the tumor microenvironment: Inflammation, counter-regulation, and tolerance. Trends Immunol..

[73] Rhim A.D., Oberstein P.E., Thomas D.H., Mirek E.T., Palermo C.F., Sastra S.A., Dekleva E.N., Saunders T., Becerra C.P., Tattersall I.W. (2014). Stromal elements act to restrain, rather than support, pancreatic ductal adenocarcinoma. Cancer Cell.

[74] Olive K.P., Jacobetz M.A., Davidson C.J., Gopinathan A., McIntyre D., Honess D., Madhu B., Goldgraben M.A., Caldwell M.E., Allard D. (2009). Inhibition of Hedgehog signaling enhances delivery of chemotherapy in a mouse model of pancreatic cancer. Science.

[75] Balachandran V.P., Łuksza M., Zhao J.N., Makarov V., Moral J.A., Remark R., Herbst B., Askan G., Bhanot U., Senbabaoglu Y. (2017). Identification of unique neoantigen qualities in long-term survivors of pancreatic cancer. Nature.

[76] Bailey P., Chang D.K., Forget M.-A., San Lucas F.A., Alvarez H.A., Haymaker C., Chattopadhyay C., Kim S.-H., Ekmekcioglu S., Grimm E.A. (2016). Exploiting the neoantigen landscape for immunotherapy of pancreatic ductal adenocarcinoma. Sci. Rep..

[77] Mahajan U.M., Langhoff E., Goni E., Costello E., Greenhalf W., Halloran C., Ormanns S., Kruger S., Boeck S., Ribback S. (2018). Immune Cell and Stromal Signature Associated With Progression-free Survival of Patients With Resected Pancreatic Ductal Adenocarcinoma. Gastroenterology.

[78] Wartenberg M., Cibin S., Zlobec I., Vassella E., Eppenberger-Castori S.M., Terracciano L., Eichmann M., Worni M., Gloor B., Perren A. (2018). Integrated genomic and immunophenotypic classification of pancreatic cancer reveals three distinct subtypes with prognostic/predictive significance. Clin. Cancer Res..

[79] Royal R.E., Levy C., Turner K., Mathur A., Hughes M., Kammula U.S., Sherry R.M., Topalian S.L., Yang J.C., Lowy I. (2010). Phase 2 trial of single agent Ipilimumab (anti-CTLA-4) for locally advanced or metastatic pancreatic adenocarcinoma. J. Immunother..

[80] Herbst R.S., Soria J.C., Kowanetz M., Fine G.D., Hamid O., Gordon M.S., Sosman J.A., McDermott D.F., Powderly J.D., Gettinger S.N. (2014). Predictive correlates of response to the anti-PD-L1 antibody MPDL3280A in cancer patients. Nature.

[81] Diaz L.A., Marabelle A., Delord J.-P., Shapira-Frommer R., Geva R., Peled N., Kim T.W., Andre T., Van Cutsem E., Guimbaud R. (2017). Pembrolizumab therapy for microsatellite instability high (MSI-H) colorectal cancer (CRC) and non-CRC. Am. Soc. Clin. Oncol..

[82] Chang L., Chang M., Chang H.M., Chang F. (2018). Microsatellite Instability: A Predictive Biomarker for Cancer Immunotherapy. Appl. Immunohistochem. Mol. Morphol. AIMM.

[83] Dudley J.C., Lin M.T., Le D.T., Eshleman J.R. (2016). Microsatellite Instability as a Biomarker for PD-1 Blockade. Clin. Cancer Res..

[84] Humphris J.L., Patch A.M., Nones K., Bailey P.J., Johns A.L., McKay S., Chang D.K., Miller D.K., Pajic M., Kassahn K.S. (2017). Hypermutation In Pancreatic Cancer. Gastroenterology.

[85] Nalley C. FDA Approves First Cancer Treatment for Any Solid Tumor with Specific Biomarker. https://journals.lww.com/oncology-times/blog/fdaactionsandupdates/pages/post.aspx?PostID=243.

[86] Overman M.J., Lopez C.D., Benson A.B., Neelapu S.S., Mettu N.B., Ko A.H., Chung V.M., Nemunaitis J.J., Reeves J.A., Bendell J.C. (2016). A randomized phase 2 study of the Bruton tyrosine kinase (Btk) inhibitor acalabrutinib alone or with pembrolizumab for metastatic pancreatic cancer (mPC). Am. Soc. Clin. Oncol..

[87] Lannutti B.J., Gulrajani M., Krantz F., Bibikova E., Covey T., Jessen K., Rothbaum W., Johnson D.M., Ulrich R. (2015). ACP-196, an orally bioavailable covalent selective inhibitor of Btk, modulates the innate tumor microenvironment, exhibits antitumor efficacy and enhances gemcitabine activity in pancreatic cancer. AACR.

[88] Hidalgo M.M., Epelbaum R., Semenisty V., Geva R., Golan T., Borazanci E.H., Stemmer S.M., Borad M.J., Park J.O., Pedersen K. (2018). Evaluation of pharmacodynamic (PD) biomarkers in patients with metastatic pancreatic cancer treated with BL-8040, a novel CXCR4 antagonist. Am. Soc. Clin. Oncol..

[89] Nesselhut J., Marx D., Lange H., Regalo G., Cillien N., Chang R.Y., Nesselhut T. (2016). Systemic treatment with anti-PD-1 antibody nivolumab in combination with vaccine therapy in advanced pancreatic cancer. Am. Soc. Clin. Oncol..

[90] Firdaus I., Waterhouse D.M., Gutierrez M., Wainberg Z.A., George B., Kelly K., Bekaii-Saab T.S., Carrizosa D.R., Soliman H.H., Fraser C.D. (2016). nab-paclitaxel (nab-P)+ nivolumab (Nivo)±gemcitabine (Gem) in patients (pts) with advanced pancreatic cancer (PC). Am. Soc. Clin. Oncol..

[91] Eriksson E., Wenthe J., Irenaeus S., Loskog A., Ullenhag G. (2016). Gemcitabine reduces MDSCs, tregs and TGFbeta-1 while restoring the teff/treg ratio in patients with pancreatic cancer. J. Transl. Med..

[92] Alvarez R., Musteanu M., Garcia-Garcia E., Lopez-Casas P., Megias D., Guerra C., Muñoz M., Quijano Y., Cubillo A., Rodriguez-Pascual J. (2013). Stromal disrupting effects of nab-paclitaxel in pancreatic cancer. Br. J. Cancer.

[93] Kunzmann V., Herrmann K., Bluemel C., Kapp M., Hartlapp I., Steger U. (2014). Intensified neoadjuvant chemotherapy with nab-paclitaxel plus gemcitabine followed by FOLFIRINOX in a patient with locally advanced unresectable pancreatic cancer. Case Rep. Oncol..

[94] Bass K.K., Mastrangelo M.J. (1998). Immunopotentiation with low-dose cyclophosphamide in the active specific immunotherapy of cancer. Cancer Immunol. Immunother. CII.

[95] Ghiringhelli F., Larmonier N., Schmitt E., Parcellier A., Cathelin D., Garrido C., Chauffert B., Solary E., Bonnotte B., Martin F. (2004). CD4+CD25+ regulatory T cells suppress tumor immunity but are sensitive to cyclophosphamide which allows immunotherapy of established tumors to be curative. Eur. J. Immunol..

[96] Ghiringhelli F., Menard C., Puig P.E., Ladoire S., Roux S., Martin F., Solary E., Le Cesne A., Zitvogel L., Chauffert B. (2007). Metronomic cyclophosphamide regimen selectively depletes CD4+CD25+ regulatory T cells and restores T and NK effector functions in end stage cancer patients. Cancer Immunol. Immunother. CII.

[97] Lord R., Nair S., Schache A., Spicer J., Somaihah N., Khoo V., Pandha H. (2007). Low dose metronomic oral cyclophosphamide for hormone resistant prostate cancer: A phase II study. J. Urol..

[98] Li X., Hu W., Zheng X., Zhang C., Du P., Zheng Z., Yang Y., Wu J., Ji M., Jiang J. (2015). Emerging immune checkpoints for cancer therapy. Acta Oncol. (Stockh. Swed.).

[99] Thomas A.M., Santarsiero L.M., Lutz E.R., Armstrong T.D., Chen Y.C., Huang L.Q., Laheru D.A., Goggins M., Hruban R.H., Jaffee E.M. (2004). Mesothelin-specific CD8(+) T cell responses provide evidence of in vivo cross-priming by antigen-presenting cells in vaccinated pancreatic cancer patients. J. Exp. Med..

[100] Lutz E.R., Wu A.A., Bigelow E., Sharma R., Mo G., Soares K., Solt S., Dorman A., Wamwea A., Yager A. (2014). Immunotherapy converts nonimmunogenic pancreatic tumors into immunogenic foci of immune regulation. Cancer Immunol. Res..

[101] Jaffee E.M., Hruban R.H., Biedrzycki B., Laheru D., Schepers K., Sauter P.R., Goemann M., Coleman J., Grochow L., Donehower R.C. (2001). Novel allogeneic granulocyte-macrophage colony-stimulating factor-secreting tumor vaccine for pancreatic cancer: A phase I trial of safety and immune activation. J. Clin. Oncol..

[102] Lutz E., Yeo C.J., Lillemoe K.D., Biedrzycki B., Kobrin B., Herman J., Sugar E., Piantadosi S., Cameron J.L., Solt S. (2011). A lethally irradiated allogeneic granulocyte-macrophage colony stimulating factor-secreting tumor vaccine for pancreatic adenocarcinoma. A Phase II trial of safety, efficacy, and immune activation. Ann. Surg..

[103] Le D.T., Whiting C.C., Lutz E.R., Nair N., Engstrom A., Lemmens E., Tagliaferri M.C., Murphy A.L., Brockstedt D.G., Jaffee E.M. (2016). Clinical and immune characteristics of rapid dropout and long-term survival in a phase II safety and efficacy study of combination CRS-207/GVAX immunotherapy in pancreatic cancer. Am. Soc. Clin. Oncol..

[104] Le D.T., Wang-Gillam A., Picozzi J., Vincent, Greten T.F., Crocenzi T.S., Springett G.M., Morse M., Zeh H., Cohen D.J., Fine R.L. (2014). A phase 2, randomized trial of GVAX pancreas and CRS-207 immunotherapy versus GVAX alone in patients with metastatic pancreatic adenocarcinoma: Updated results. Am. Soc. Clin. Oncol..

[105] Le D.T., Ko A.H., Wainberg Z.A., Picozzi V.J., Kindler H.L., Wang-Gillam A., Oberstein P.E., Morse M., Zeh H., Weekes C.D. (2017). Results from a phase 2b, randomized, multicenter study of GVAX pancreas and CRS-207 compared to chemotherapy in adults with previously-treated metastatic pancreatic adenocarcinoma (ECLIPSE Study). J. Clin. Oncol..

[106] Joziasse D., Oriol R. (1999). Xenotransplantation: The importance of the Galα1, 3Gal epitope in hyperacute vascular rejection. Biochim. Biophys. Acta (BBA) Mol. Basis Dis..

[107] Hardacre J.M., Mulcahy M., Small W., Talamonti M., Obel J., Krishnamurthi S., Rocha-Lima C.S., Safran H., Lenz H.-J., Chiorean E.G. (2013). Addition of algenpantucel-L immunotherapy to standard adjuvant therapy for pancreatic cancer: A phase 2 study. J. Gastrointest. Surg..

[108] Thind K., Padrnos L.J., Ramanathan R.K., Borad M.J. (2017). Immunotherapy in pancreatic cancer treatment: A new frontier. Ther. Adv. Gastroenterol..

[109] Apostolopoulos V., Xing P.-X., McKenzie I.F. (1994). Murine immune response to cells transfected with human MUC1: Immunization with cellular and synthetic antigens. Cancer Res..

[110] Weiss S., Bogen B. (1991). MHC class II—Restricted presentation of intracellular antigen. Cell.

[111] Jardetzky T., Lane W., Robinson R., Madden D., Wiley D. (1991). Identification of self peptides bound to purified HLA-B27. Nature.

[112] Abrams S.I., Khleif S.N., Bergmann-Leitner E.S., Kantor J.A., Chung Y., Hamilton J.M., Schlom J. (1997). Generation of Stable CD4+ and CD8+ T Cell Lines from Patients Immunized withrasOncogene-Derived Peptides Reflecting Codon 12 Mutations. Cell. Immunol..

[113] Cereda V., Vergati M., Huen N.-Y., di Bari M.G., Jochems C., Intrivici C., Gulley J.L., Apelian D., Schlom J., Tsang K.Y. (2011). Maturation of human dendritic cells with Saccharomyces cerevisiae (yeast) reduces the number and function of regulatory T cells and enhances the ratio of antigen-specific effectors to regulatory T cells. Vaccine.

[114] Gjertsen M.K., Breivik J., Saeterdal I., Thorsby E., Gaudernack G., Bakka A., Soøreide O., Solheim B. (1995). Vaccination with mutant ras peptides and induction of T-cell responsiveness in pancreatic carcinoma patients carrying the corresponding RAS mutation. Lancet.

[115] Gjertsen M.K., Buanes T., Rosseland A.R., Bakka A., Gladhaug I., Søreide O., Eriksen J.A., Møller M., Baksaas I., Lothe R.A. (2001). Intradermal ras peptide vaccination with granulocyte-macrophage colony-stimulating factor as adjuvant: Clinical and immunological responses in patients with pancreatic adenocarcinoma. Int. J. Cancer.

[116] Carbone D.P., Ciernik I.F., Kelley M.J., Smith M.C., Nadaf S., Kavanaugh D., Maher V.E., Stipanov M., Contois D., Johnson B.E. (2005). Immunization with mutant p53-and K-ras-derived peptides in cancer patients: Immune response and clinical outcome. J. Clin. Oncol..

[117] Toubaji A., Achtar M., Provenzano M., Herrin V.E., Behrens R., Hamilton M., Bernstein S., Venzon D., Gause B., Marincola F. (2008). Pilot study of mutant ras peptide-based vaccine as an adjuvant treatment in pancreatic and colorectal cancers. Cancer Immunol. Immunother. CII.

[118] Abou-Alfa G.K., Chapman P.B., Feilchenfeldt J., Brennan M.F., Capanu M., Gansukh B., Jacobs G., Levin A., Neville D., Kelsen D.P. (2011). Targeting mutated K-ras in pancreatic adenocarcinoma using an adjuvant vaccine. Am. J. Clin. Oncol..

[119] Kyte J.A., Gaudernack G., Dueland S., Trachsel S., Julsrud L., Aamdal S. (2011). Telomerase peptide vaccination combined with temozolomide: A clinical trial in stage IV melanoma patients. Clin. Cancer Res..

[120] Bernhardt S.L., Gjertsen M.K., Trachsel S., Moller M., Eriksen J.A., Meo M., Buanes T., Gaudernack G. (2006). Telomerase peptide vaccination of patients with non-resectable pancreatic cancer: A dose escalating phase I/II study. Br. J. Cancer.

[121] Prasad N.B., Biankin A.V., Fukushima N., Maitra A., Dhara S., Elkahloun A.G., Hruban R.H., Goggins M., Leach S.D. (2005). Gene expression profiles in pancreatic intraepithelial neoplasia reflect the effects of Hedgehog signaling on pancreatic ductal epithelial cells. Cancer Res..

[122] Smith J.P., Shih A., Wu Y., McLaughlin P.J., Zagon I.S. (1996). Gastrin regulates growth of human pancreatic cancer in a tonic and autocrine fashion. Am. J. Physiol..

[123] Berna M.J., Seiz O., Nast J.F., Benten D., Blaker M., Koch J., Lohse A.W., Pace A. (2010). CCK1 and CCK2 receptors are expressed on pancreatic stellate cells and induce collagen production. J. Biol. Chem..

[124] Brett B.T., Smith S.C., Bouvier C.V., Michaeli D., Hochhauser D., Davidson B.R., Kurzawinski T.R., Watkinson A.F., Van Someren N., Pounder R.E. (2002). Phase II study of anti-gastrin-17 antibodies, raised to G17DT, in advanced pancreatic cancer. J. Clin. Oncol..

[125] Gilliam A.D., Broome P., Topuzov E.G., Garin A.M., Pulay I., Humphreys J., Whitehead A., Takhar A., Rowlands B.J., Beckingham I.J. (2012). An international multicenter randomized controlled trial of G17DT in patients with pancreatic cancer. Pancreas.

[126] Shapiro J., Marshall J., Karasek P., Figer A., Oettle H., Couture F., Jeziorski K., Broome P., Hawkins R. (2005). G17DT+ gemcitabine [Gem] versus placebo+ Gem in untreated subjects with locally advanced, recurrent, or metastatic adenocarcinoma of the pancreas: Results of a randomized, double-blind, multinational, multicenter study. J. Clin. Oncol..

[127] Suzuki H., Onishi H., Wada J., Yamasaki A., Tanaka H., Nakano K., Morisaki T., Katano M. (2010). VEGFR2 is selectively expressed by FOXP3high CD4+ Treg. Eur. J. Immunol..

[128] Terme M., Tartour E., Taieb J. (2013). VEGFA/VEGFR2-targeted therapies prevent the VEGFA-induced proliferation of regulatory T cells in cancer. Oncoimmunology.

[129] Fricke I., Mirza N., Dupont J., Lockhart C., Jackson A., Lee J.H., Sosman J.A., Gabrilovich D.I. (2007). Vascular endothelial growth factor-trap overcomes defects in dendritic cell differentiation but does not improve antigen-specific immune responses. Clin. Cancer Res..

[130] Horikawa N., Abiko K., Matsumura N., Hamanishi J., Baba T., Yamaguchi K., Yoshioka Y., Koshiyama M., Konishi I. (2017). Expression of Vascular Endothelial Growth Factor in Ovarian Cancer Inhibits Tumor Immunity through the Accumulation of Myeloid-Derived Suppressor Cells. Clin. Cancer Res..

[131] Miyazawa M., Ohsawa R., Tsunoda T., Hirono S., Kawai M., Tani M., Nakamura Y., Yamaue H. (2010). Phase I clinical trial using peptide vaccine for human vascular endothelial growth factor receptor 2 in combination with gemcitabine for patients with advanced pancreatic cancer. Cancer Sci..

[132] Long A.H., Highfill S.L., Cui Y., Smith J.P., Walker A.J., Ramakrishna S., El-Etriby R., Galli S., Tsokos M., Orentas R.J. (2016). Reduction of MDSCs with all-trans retinoic acid improves CAR therapy efficacy for sarcomas. Cancer Immunol. Res..

[133] Ene–Obong A., Clear A.J., Watt J., Wang J., Fatah R., Riches J.C., Marshall J.F., Chin–Aleong J., Chelala C., Gribben J.G. (2013). Activated pancreatic stellate cells sequester CD8+ T cells to reduce their infiltration of the juxtatumoral compartment of pancreatic ductal adenocarcinoma. Gastroenterology.

[134] Froeling F.E., Feig C., Chelala C., Dobson R., Mein C.E., Tuveson D.A., Clevers H., Hart I.R., Kocher H.M. (2011). Retinoic acid–induced pancreatic stellate cell quiescence reduces paracrine Wnt–β-catenin signaling to slow tumor progression. Gastroenterology.

[135] Schwartz G.G., Eads D., Naczki C., Northrup S., Chen T., Koumenis C. (2008). 19-nor-1α, 25-Dihydroxyvitamin D2 (Paricalcitol) inhibits the proliferation of human pancreatic cancer cells in vitro and in vivo. Cancer Biol. Ther..

[136] Apte M.V., Wilson J.S. (2012). Dangerous liaisons: Pancreatic stellate cells and pancreatic cancer cells. J. Gastroenterol. Hepatol..

[137] Wang-Gillam A., Lockhart A., Tan B., Suresh R., Lim K.-H., Ratner L., Morton A., Huffman J., Marquez S., Boice N. (2018). Phase I study of defactinib combined with pembrolizumab and gemcitabine in advanced cancer. J. Clin. Oncol..

[138] Jiang H., Hegde S., Knolhoff B.L., Zhu Y., Herndon J.M., Meyer M.A., Nywening T.M., Hawkins W.G., Shapiro I.M., Weaver D.T. (2016). Targeting focal adhesion kinase renders pancreatic cancers responsive to checkpoint immunotherapy. Nat. Med..

[139] Dammeijer F., Lievense L.A., Kaijen-Lambers M.E., van Nimwegen M., Bezemer K., Hegmans J.P., van Hall T., Hendriks R.W., Aerts J.G. (2017). Depletion of Tumor-Associated Macrophages with a CSF-1R Kinase Inhibitor Enhances Antitumor Immunity and Survival Induced by DC Immunotherapy. Cancer Immunol. Res..

[140] Zboralski D., Kruschinski A., Eulberg D., Vater A. (2016). CXCL12 inhibition with NOX-A12 (olaptesed pegol) increases T and NK cell infiltration and synergizes with immune checkpoint blockade in tumour-stroma spheroids. Ann. Oncol..

[141] Scotton C.J., Wilson J.L., Scott K., Stamp G., Wilbanks G.D., Fricker S., Bridger G., Balkwill F.R. (2002). Multiple actions of the chemokine CXCL12 on epithelial tumor cells in human ovarian cancer. Cancer Res..

[142] Orimo A., Gupta P.B., Sgroi D.C., Arenzana-Seisdedos F., Delaunay T., Naeem R., Carey V.J., Richardson A.L., Weinberg R.A. (2005). Stromal fibroblasts present in invasive human breast carcinomas promote tumor growth and angiogenesis through elevated SDF-1/CXCL12 secretion. Cell.

[143] Hamdan R., Zhou Z., Kleinerman E.S. (2014). Blocking SDF-1α/CXCR4 Downregulates PDGF-B and Inhibits Bone Marrow–Derived Pericyte Differentiation and Tumor Vascular Expansion in Ewing Tumors. Mol. Cancer Ther..

[144] Dredge K., Horsfall R., Robinson S.P., Zhang L.-H., Lu L., Tang Y., Shirley M.A., Muller G., Schafer P., Stirling D. (2005). Orally administered lenalidomide (CC-5013) is anti-angiogenic in vivo and inhibits endothelial cell migration and Akt phosphorylation in vitro. Microvasc. Res..

[145] Wu L., Parton A., Lu L., Adams M., Schafer P., Bartlett J.B. (2011). Lenalidomide enhances antibody-dependent cellular cytotoxicity of solid tumor cells in vitro: Influence of host immune and tumor markers. Cancer Immunol. Immunother..

[146] Ullenhag G.J., Mozaffari F., Broberg M., Mellstedt H., Liljefors M. (2017). Clinical and immune effects of lenalidomide in combination with gemcitabine in patients with advanced pancreatic cancer. PLoS ONE.

[147] Corral L.G., Haslett P.A., Muller G.W., Chen R., Wong L.-M., Ocampo C.J., Patterson R.T., Stirling D.I., Kaplan G. (1999). Differential cytokine modulation and T cell activation by two distinct classes of thalidomide analogues that are potent inhibitors of TNF-α. J. Immunol..

[148] Schlingensiepen K.H., Jaschinski F., Lang S.A., Moser C., Geissler E.K., Schlitt H.J., Kielmanowicz M., Schneider A. (2011). Transforming growth factor-beta 2 gene silencing with trabedersen (AP 12009) in pancreatic cancer. Cancer Sci..

[149] Oettle H., Seufferlein T., Luger T., Schmid R.M., von Wichert G., Endlicher E., Garbe C., Kaehler K.K., Enk A., Schneider A. (2012). Final results of a phase I/II study in patients with pancreatic cancer, malignant melanoma, and colorectal carcinoma with trabedersen. Am. Soc. Clin. Oncol..

[150] Hecht J.R., Bedford R., Abbruzzese J.L., Lahoti S., Reid T.R., Soetikno R.M., Kirn D.H., Freeman S.M. (2003). A phase I/II trial of intratumoral endoscopic ultrasound injection of ONYX-015 with intravenous gemcitabine in unresectable pancreatic carcinoma. Clin. Cancer Res..

[151] Mulvihill S., Warren R., Venook A., Adler A., Randlev B., Heise C., Kirn D. (2001). Safety and feasibility of injection with an E1B-55 kDa gene-deleted, replication-selective adenovirus (ONYX-015) into primary carcinomas of the pancreas: A phase I trial. Gene Ther..

[152] Melisi D., Garcia-Carbonero R., Macarulla T., Pezet D., Deplanque G., Fuchs M., Trojan J., Oettle H., Kozloff M., Cleverly A. (2016). A phase II, double-blind study of galunisertib+ gemcitabine (GG) vs. gemcitabine+ placebo (GP) in patients (pts) with unresectable pancreatic cancer (PC). Am. Soc. Clin. Oncol..

[153] Feldmann G., Fendrich V., McGovern K., Bedja D., Bisht S., Alvarez H., Koorstra J.-B.M., Habbe N., Karikari C., Mullendore M. (2008). An orally bioavailable small-molecule inhibitor of Hedgehog signaling inhibits tumor initiation and metastasis in pancreatic cancer. Mol. Cancer Ther..

[154] Goldman J., Eckhardt S.G., Borad M.J., Curtis K.K., Hidalgo M., Calvo E., Ryan D.P., Wirth L.J., Parikh A., Partyka J. (2015). Phase 1 dose-escalation trial of the oral investigational hedgehog signaling pathway inhibitor TAK-441 in patients with advanced solid tumors. Clin. Cancer Res..

[155] Guba S., Mukhopadhyay S., Desaiah D., Andre V. (2016). A phase 1b/2 dose escalation and cohort expansion study of the safety, tolerability and efficacy of a transforming growth factor-beta (TGF-β) receptor I kinase inhibitor (galunisertib) in combination with anti–PD-1 (nivolumab) in advanced refractory solid tumours. Ann. Oncol..

[156] Bogdahn U., Hau P., Stockhammer G., Venkataramana N., Mahapatra A., Suri A.A., Balasubramaniam A., Nair S., Oliushine V., Parfenov V. (2010). Targeted therapy for high-grade glioma with the TGF-β2 inhibitor trabedersen: Results of a randomized and controlled phase IIb study. Neuro-Oncology.

